# Direct observation of CD4 T cell morphologies and their cross-sectional traction force derivation on quartz nanopillar substrates using focused ion beam technique

**DOI:** 10.1186/1556-276X-8-332

**Published:** 2013-07-23

**Authors:** Dong-Joo Kim, Gil-Sung Kim, Jung-Hwan Hyung, Won-Yong Lee, Chang-Hee Hong, Sang-Kwon Lee

**Affiliations:** 1 Basic Research Laboratory (BRL), Department of Semiconductor Science and Technology, Chonbuk National University, Jeonju 561-756, Republic of Korea; 2 Department of Physics, Chung-Ang University, Seoul 156-756, Republic of Korea

**Keywords:** Cell traction force, Cell adhesion, CD4 T cell, Cell migration, Focused ion beam

## Abstract

Direct observations of the primary mouse CD4 T cell morphologies, e.g., cell adhesion and cell spreading by culturing CD4 T cells in a short period of incubation (e.g., 20 min) on streptavidin-functionalized quartz nanopillar arrays (QNPA) using a high-content scanning electron microscopy method were reported. Furthermore, we first demonstrated cross-sectional cell traction force distribution of surface-bound CD4 T cells on QNPA substrates by culturing the cells on top of the QNPA and further analysis in deflection of underlying QNPA via focused ion beam-assisted technique.

## Background

Cell adhesion is the initial step upon interactions of substrate materials with loaded cells. In particular, it was shown that nanotopography influences diverse cell behaviors such as cell adhesion, cytoskeletal organization, apoptosis, macrophage activation, and gene expression [1,2], which in turn leads to proliferation, differentiation, and migration on various nanostructures including nanofibers [3], nanopillars [4], and nanogrooves [5,6]. As a result, cell behaviors are critically determined by the interaction between nanoscale cellular surface components such as microvilli, filopodia, extracellular matrix (ECM), and the underlying nanostructure topography [7]. However, little is known of how the use of size and shape-matched diverse nanometer-scale topographies interact to not only the forthcoming cells but also the nanoscale cellular surface components of cells bound on the nanotopographic substrates in cell adhesion steps even at the very early stage of incubation (<20 min).

Cell traction force (CTF) is crucial to cell migration, proliferation, differentiation, cell shape maintenance, mechanical cell-signal generation, and other cellular functions just following adhesion step on the nanotopographic substrates. Once transmitted to the ECM through stress fibers via focal adhesions, which are assemblies of ECM proteins, transmembrane receptor, and cytoplasmic structural and signaling proteins (e.g., integrins), CTF directs many cellular functions [8]. In addition, CTF plays an important role in many biological processes such as inflammation [9], wound healing [10], angiogenesis [11], and cancer metastasis [12]. Thus, a complete knowledge of CTF regulation and the improvement of the ability to measure CTFs are currently critical in clear understating physiological and pathological events at both the tissue and organ levels. To date, various techniques have been developed and have refined over the years to measure CTFs of single cells or population of cells, including cell-populated collagen gel method [13], micromechanical cantilever beam-based force sensor array [14], cell traction force microscopy [15], and elastomeric micropost array [16,17]. In 2009, Li et al. reported another favorable method to quantify the traction force of a single cell by aligned silicon nanowire (SiNW) arrays [18]. They reported that the CTFs of the cells cultured on this SiNW arrays could be calculated from these underlying SiNW deflections. However, no further lateral CTF information (cross-sectional) inside the cell underlying on the nanotopographic substrates was provided.

In this letter, we first report on direct observations of the primary mouse CD4 T cell morphologies by culturing CD4 T cells on streptavidin (STR)-functionalized quartz nanopillar arrays (QNPA) using a scanning electron microscopy (SEM) method and then demonstrate a new alternative technique to measure cross-sectional cell traction force distribution of surface-bound CD4 T cells including those inside the cells on QNPA substrates by culturing the cells on the top of the QNPA and further analysis in deflection of underlying QNPA via focused ion beam (FIB)-assisted technique. It conducted both a high-performance etching and imaging scheme from FIB and finite element method (FEM)-based computer simulation tools with well-defined QNPA substrates. We suggest that the use of the FIB-based technique combined with QNPA and FEM simulation would be a powerful and fine process to evaluate cross-sectional CTFs of single cells.

## Methods

**Figure 1:**
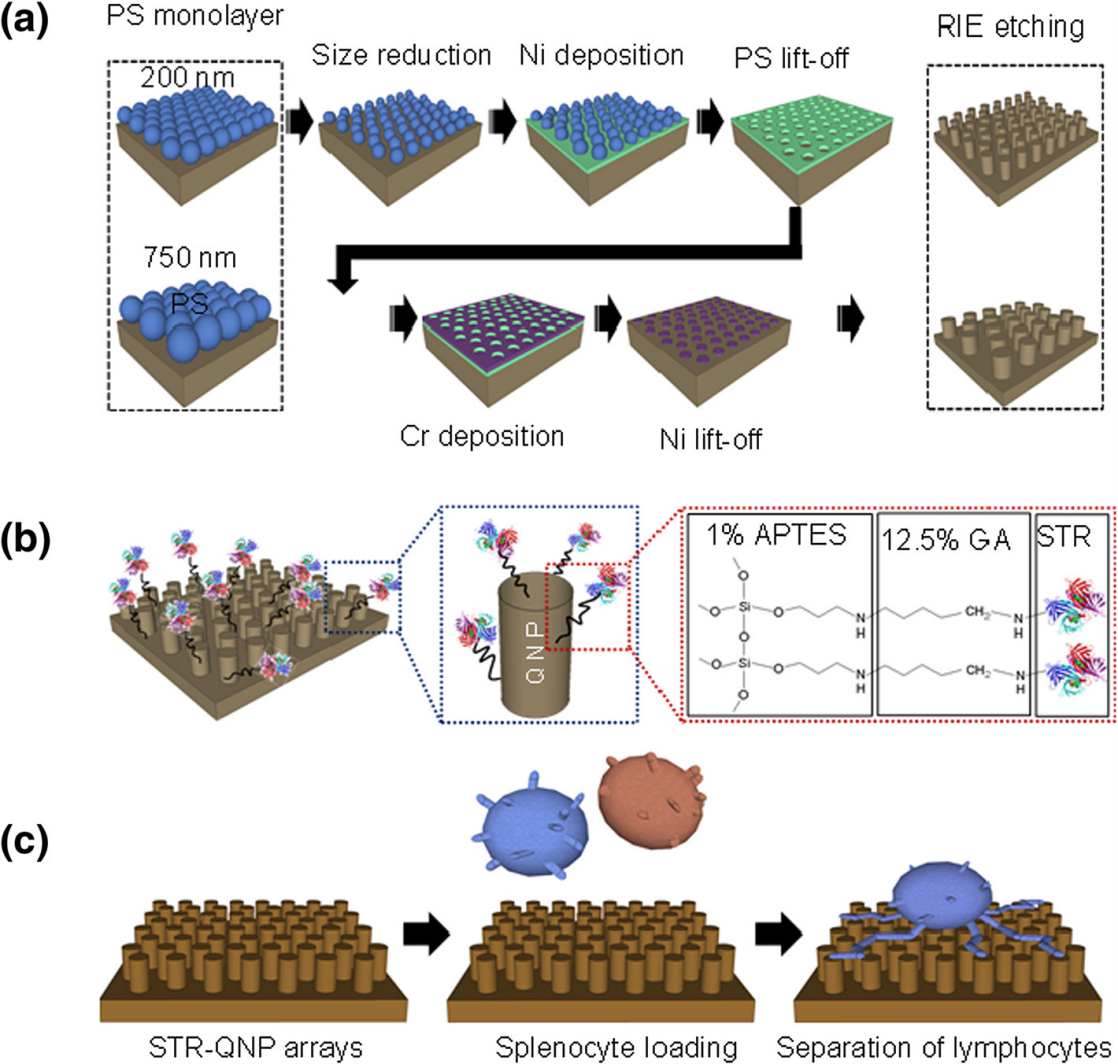
**Schematic diagram of QNPA fabrication and separation processes. (a)** Schematic diagram outlining the fabrication of quartz nanopillar arrays (QNPAs) where two different sizes of PS were presented for specific example. **(b) **Surface functionalization including APTES, GA, and STR reactions of QNPAs on a quartz substrate. **(c)** Schematic diagram of specific CD4 T cell separation process from introduced cell suspension containing CD4 T, CD8 T, NK, and NKT cells from primary mouse splenocytes.

Figure 1a,b shows a schematic illustration of QNPA fabrication processes and further surface functionalization processes, respectively. First, the fabrication process went through a series of process including polystyrene (PS) monolayer deposition, PS size reduction, Ni metal deposition, PS lift-off, additional Cr metal deposition, Ni lift-off, and final reactive ion etching process we have improved previously [19,20]. In addition, the surface of QNPA substrates treated by O_2_ plasma was then applied by three-step surface functionalization processes using 1% (*v*/*v*) (3-aminopropyl)-triethoxysilane (APTES) in ethanol for 30 min at room temperature, 12.5% (*v*/*v*) glutaraldehyde (GA) in distilled water for 4 h on a 2D rocker, and approximately 50-μg/mL STR solution in phosphate buffered saline (PBS) overnight in an incubator (37°C, 5% CO_2_). We used this surface-functionalized method on nanotopographic substrates to separate targeting specific cells (e.g., CD4 T cells) among different kinds of cells via the novel STR-biotin conjugation technique to capture the incoming targeting cells in PBS solution as we have developed previously [20,21]. The T lymphocytes were mouse CD4 T cells from whole mouse splenocytes. Mouse splenocytes (approximately 10^5^ cells per sample) containing CD4 T, CD8 T, natural killer (NK), and natural killer T (NKT) cells were prepared from the spleen of C57BL/6/mice (Nara Biotech, Seoul, South Korea) [22]. Prior to introducing the cell suspension in PBS solution onto the QNPA substrates (0.7 cm × 0.7 cm), the cell population (Figure 1c) with a final volume of approximately 30 μl was first reacted with biotin anti-mouse CD4 antibody and incubated at 4°C for 20 min. The cell suspension containing T cells and other cells pre-reacted with biotin anti-mouse CD4 antibody was then introduced on the STR-functionalized QNPA substrates. Following 20 min of incubation at 4°C in a refrigerator, where the CD4 T cells were in a very early stage of cell adhesion on the QNPA substrates, unbound cells were removed by rinsing with PBS solution. This step was repeated at least five times for 10 min on a 2D rocker to completely remove nonspecifically unbound cells from the QNPA substrates (third image in Figure 1c). Our experiments were focused on targeted CD4 T cell adhesion on STR-functionalized QNPA substrates at a very early stage of cell adhesion (<20 min). To examine the morphologies of the captured CD4 T cells bound on STR-conjugated QNPA substrates, SEM observation was performed. For the SEM observation of the captured cells on QNPA substrate, a series of cell-fixing processes are required as follows. The T cells were first fixed with 4% GA in the refrigerator for 2 h, followed by a post-fix process using 1% osmium tetroxide for 2 h. The T cells were then dehydrated through a series of ethanol concentrations (25%, 50%, 75%, 95%, and 100%) and slowly dried at vacuum-connected desiccators for 24 h [21,23,24]. According to a previous report, the average conventional fixed material, after all steps of preservation, retained 72% of its initial size [25]. Once the samples were dry in the desiccators, the surface-bound T cells were sputter-coated with platinum before the SEM measurement was performed.

## Results and discussion

**Figure 2:**
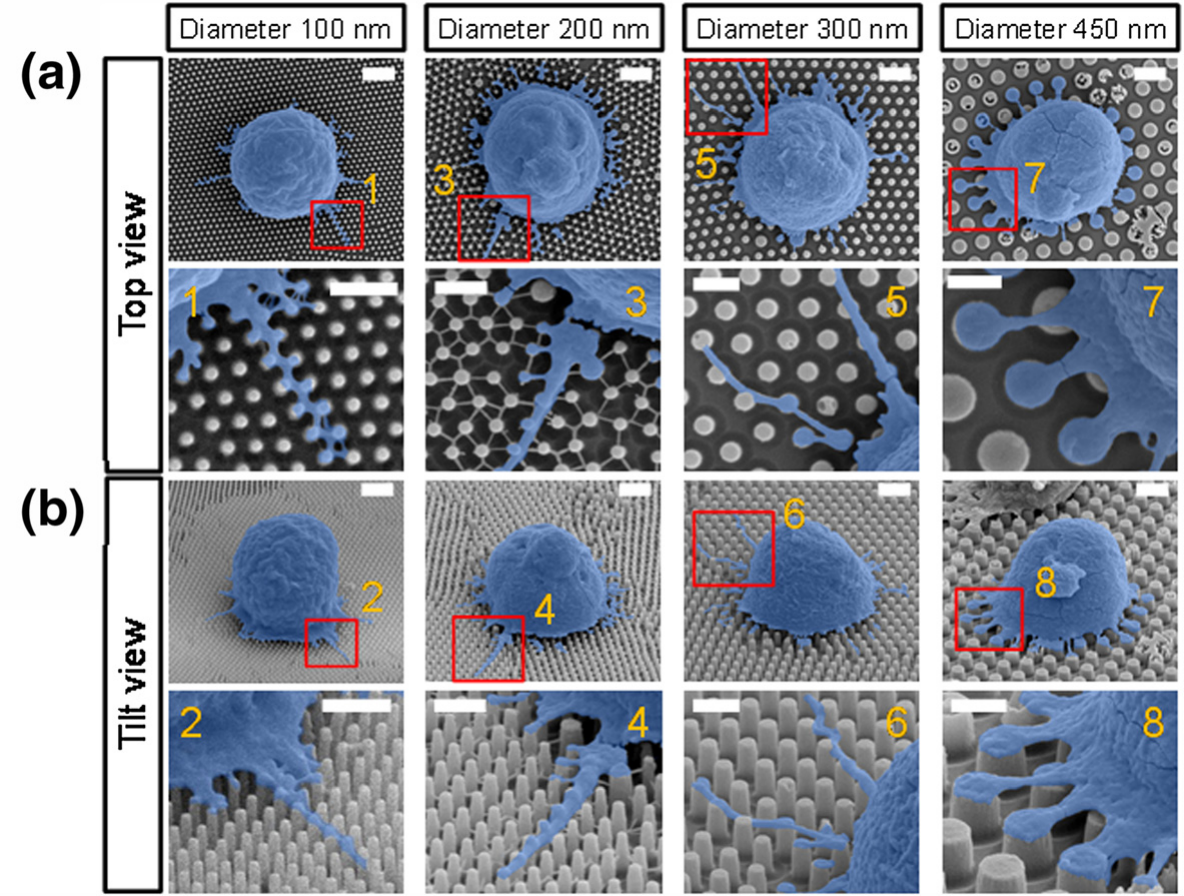
**SEM images of captured CD4 T cells on four different sizes of QNPA substrates. (a)** Top and **(b)** tilt views. All captured cells were highlighted in blue for easy distinction.

**Figure 3:**
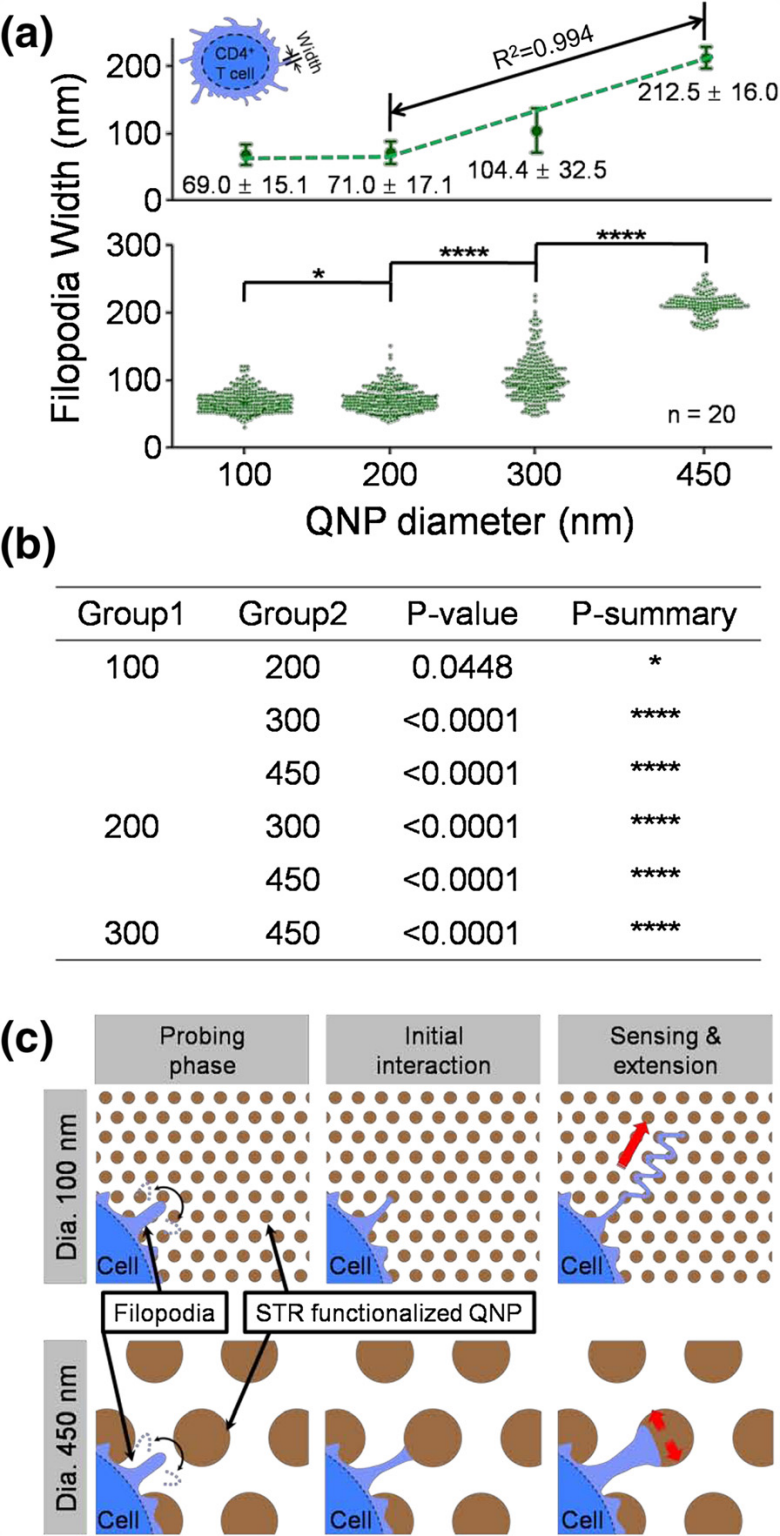
**Filopodial width distribution, *p *values, and diagram of CD4 cells bound on four QNPA substrates. (a)** Filopodial width distribution of CD4 cells bound on the four different STR-functionalized QNPA substrates after only 20 min of incubation at 4°C. Selected filopodia with distribution (top part of figure) in which only approximately 80% of filopodial width taken from the results (bottom part of figure). **(b)** Summary of *p* values for filopodial width distribution of captured CD4 T cells on four different QNPA substrates. *p* values <0.0001 (****) are considered statistically significant. Less significant statistical difference is represented (* *p* = 0.0448). **(c)** Schematic diagram of CD4 T cell spreading mechanism just for 20 min of incubation.

Figure 2a,b shows SEM images (top, tilt, and enlarged views) of CD4 T cells bound on four different sizes of STR-functionalized QNPA substrates. The diameters of QNPA using four PS NPs (200, 300, 430, and 750 nm in diameter) were approximately 100, 200, 300, and 450 nm, respectively, as determined by SEM. The detailed morphologies of the captured T cells on STR-QNPA substrates were examined by quantitative SEM analysis using a cell freezing technique described previously. These results exhibit that the captured T cells were well bound on the surface with different morphologies of filopodia or lamellipodia as shown in Figure 2a,b. Interestingly, these images indicate that the morphology (e.g., width of these surface components) of the captured T cells is highly correlated with the size of QNPA in diameter from 200 to 450 nm. To ensure the evaluation of the filopodial width in the early stage of cell adhesion, we quantified at least approximately 20 cells. As a result, the widths of filopodia protruding from T cells bound on QNPA were determined to be approximately 69.00 ± 15.10, 71.60 ± 17.1, 104.40 ± 32.50, and 212.50 ± 16.00 nm corresponding to QNPA surface diameters of approximately 100, 200, 300, and 450 nm, respectively, as shown in Figures 2 and 3a. Filopodial morphologies on STR-QNPA below approximately 300 nm in diameter present a long extended shape, but it extends to be remarkably narrow as it has to be confined by adjacent STR-QNPs with 450 nm diameter. We noticed that captured CD4 T cells on the STR-QNPA surfaces exhibited striking differences in morphology on the varied diameters, even under the condition of extremely early stages of adhesion and statically stable activity of T cells (approximately 20-min incubation at 4°C). Furthermore, to assess the significance of our correlation results, *p* values were calculated with neighboring column data. Figure 3a exhibits that the distribution of extended filopodial width of the captured CD4 T cells were observed to increase in width by increasing the diameter of QNPA from 200 to 450 nm (**** *p* < 0.0001, Figure 3b,c), resulting in a good linear response between the width of T cells and diameter of QNPA (*R*^2^ = 0.994, *n* = 20). On the other hand, the filopodial width for 100-nm QNPA shows a similar trend in size to that of the 200-nm QNPA, exhibiting a statistically insignificant difference (* *p* = 0.0448, bottom part in Figure 3a,b).

These results suggest that the microvilli (filopodia or lamellipodia) of CD4 T cells closely react with the QNPA substrates via high-affinity STR-biotin conjugation as we have proven previously [22] and extend filopodia of widths depending on the diameter of the QNPAs to identify the size of the structures underneath the cells using filopodia as illustrated in Figure 3c. This strong linear response in the filopodia extending from the T cells bound on the solid-state surfaces with the nanopillar diameters of the surface could be explained by a contact guidance phenomenon. This is usually used to explain the behavior of fibroblast filopodia on nanostructured substrates with long incubation [5,26,27]. According to the contact guidance phenomenon, the T cells extend the filopodia to recognize and sense the surface features of nanotopographic substrates when they are bound on the surface at the early state of the adhesion and then form themselves on the substrates with a similar size of the nanostructure underneath the cells (Figure 3c). Our observation corresponds well with previous results from Dalby et al. [28] even if we conducted it on T cells instead of epithelial cell line.

**Figure 4:**
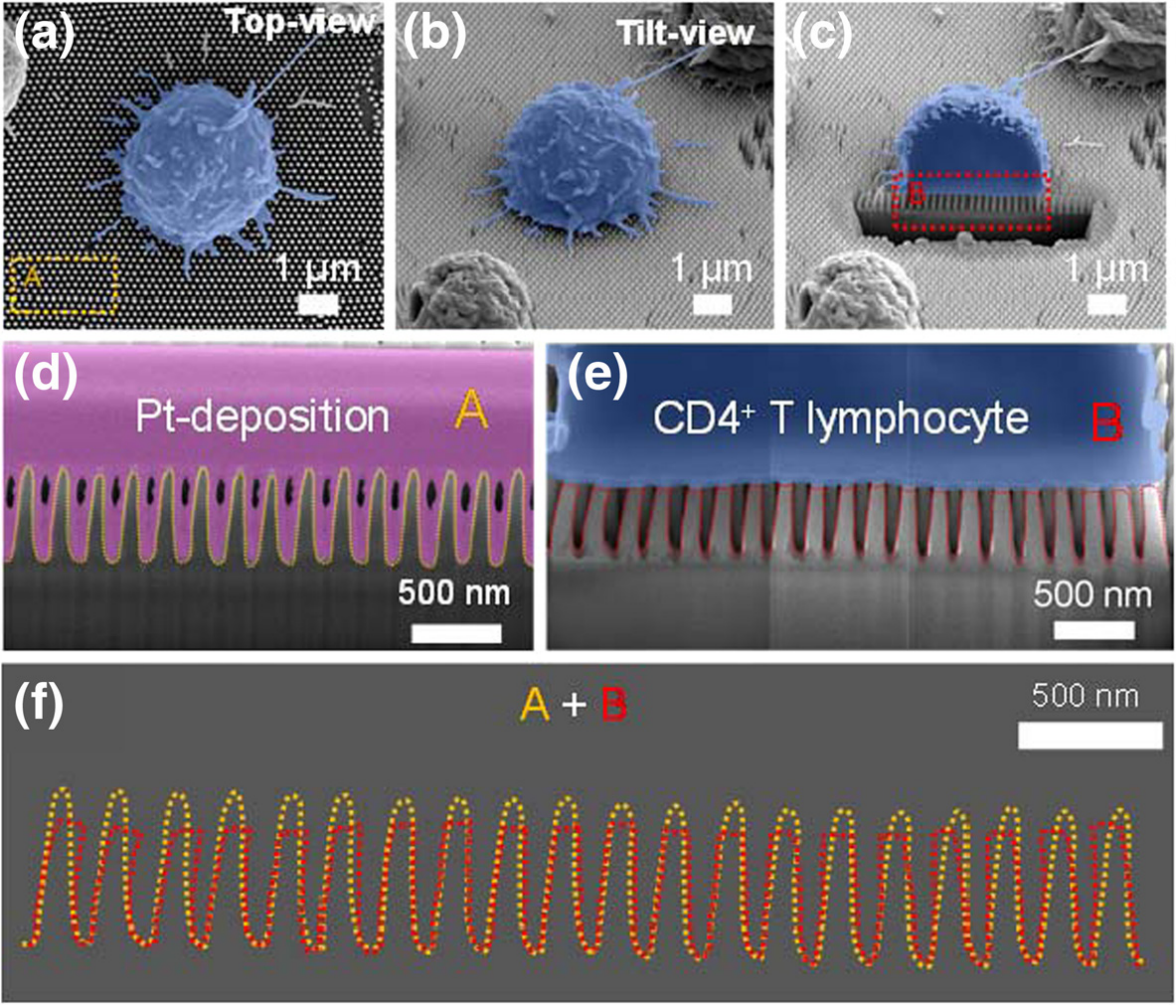
**SEM images of the CD4 T cell and QNPA. (a, b, c)** SEM images (top and tilt views) of the CT4 T cell on the QNPA substrates before and after FIB ion milling, respectively. **(d, e)** Cross-sectional SEM images of QNPA without and with surface-bound T cell, respectively. **(f)** Overlapped images of QNPA from only QNPA and from QNPA covered by the cell. All cells were highlighted in blue, while the Pt was in purple, for clear differentiation.

**Figure 5:**
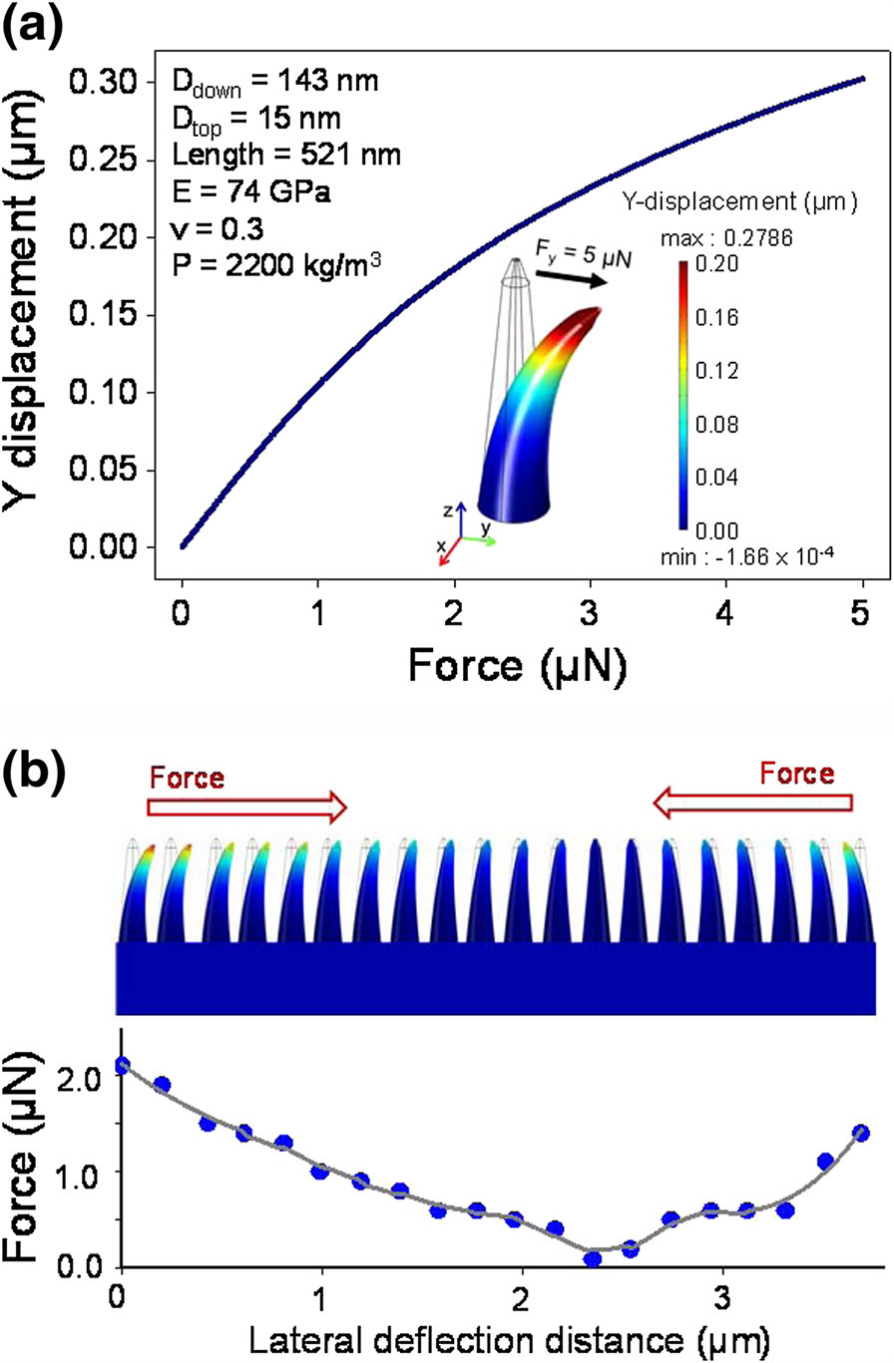
**Relationship between lateral deflection distance and CTFs and cross-sectional CTF distribution of CD4 T cells. (a)** The relationship between the lateral deflection distance (*y* displacement) and CTFs of the CD4 T cell acting on the QNPA substrates using nonlinear model in COMSOL Multiphysics® software. **(b)** Cross-sectional CTF distribution of the CD4 T cell on STR-QNPA substrates, exhibiting that the CTFs at the edge of the cells are much stronger than those at the center part of the cells.

To investigate cross-sectional CTF of T cells on STR-functionalized QNPA substrate, we utilized both a high-performance etching and imaging scheme from FIB and FEM-based commercial simulation tools. In this regard, we first carried out the cross-sectional etching of the surface-bound T cells on QNPA substrates to assure CTFs exerted on the T cells. Figure 4a,b,c shows SEM images (top, tilt, and cross-sectional views) of the cell on the QNPA substrates before and after Ga^+^ ion milling process of dehydrated CD4 T cell using FIB technique, respectively. These figures show that the captured T cells on STR-functionalized QNPA were securely bound on the surface of QNPA. In addition, to further evaluate the deflection of the QNPA shown in Figure 4e, we took cross-sectional images both from only QNPA substrate (‘A’ region in Figure 4a) and from the CD4 T cell bound on the QNPA (‘B’ region in Figure 4c) as shown in Figure 4d,e, respectively (enlarged images of the cross-sectional views). This result exhibits that each nanopillar was clearly bended to the center region as shown in the overlapped images (Figure 4f). Accordingly, we can straightforwardly extract the deflection distance of each nanopillar, which is the key parameter to derive the CTFs with FEM simulation, from the SEM observation. According to the maximum bending distance (*x*) and the corresponding bending force (*f*) [18,29]*f* = (3*EI* / *L*^3^)*x*, where *E* is the elastic modulus of quartz nanopillar, *I* is the area moment of inertia, *L* is the height of the nanopillar, and *x* is the bending distance, the CTF (*f*) required to bend a nanopillar can be derived from the lateral displacement (*x*) of a nanopillar parallel to the quartz substrate. For this purpose, we then carried out FEM simulations using commercial COMSOL Multiphysics® (COMSOL AB, Stockholm, Sweden) software using the experimental measurements from the SEM observation and mechanical properties of the quartz nanopillar. Using a nonlinear model in COMSOL Multiphysics® software, we derived the relationship, which is served for the calibration to quantify the CTF of the cells, between the lateral deflection distance and CTFs of the CD4 T cell acting on the QNPA substrates as shown in Figure 5a. As a result, Figure 5b shows the cross-sectional CTF distribution of the CD4 T cell on STR-QNPA substrates, exhibiting that the CTFs at the edge of the cells are much stronger than those at center part of the cells. The values of CTFs for the captured CD4 T cells on STR-functionalized QNPA substrates are determined to be in the range of 0.1 to 2.1 μN, while the deflection distances were determined to be 0.2 to 3.69 μm, just after 20 min of incubation. Li et al. reported that the CTFs between the L929 cells and silicon nanowire arrays were in the range of 2.7~4.3 μN when cultured for 2 to 36 h, which is 1.3~1.6 times higher in CTFs as compared to our observation in maximum CTFs of CD4 T cells on QNPA substrates [18]. Our previous results [23] suggested that the traction force on the nanostructured substrates increased with increasing incubation times, which is in good agreement with previous results in cell migration with an increase in culture times [18]. As a result, the values of CTFs of the captured CD4 T cell on STR-functionalized QNPA substrate with short periods of incubation (<20 min) are much lower than those from other cells for long periods of incubation (>30 h).

## Conclusions

In conclusion, we have studied the behaviors (e.g., cell adhesion and spreading) of CD4 T cells captured on STR-functionalized QNPA substrates at the very early stage of incubation (less than 20 min). For this study, we prepared four different sizes of QNPA substrates using a modified self-assembly method. On the basis of our results, we found that the distribution of extended filopodial width of the captured CD4 T cells was highly related to the diameter of QNPA (200 to 450 nm), indicating that extended filopodia of CD4 T cells increased in width with the increasing diameter of QNPA from 200 to 450 nm. Furthermore, we demonstrated cross-sectional CTF distribution of surface-bound CD4 T cells on QNPA substrates by culturing the cells on the tip of the QNPA and further analysis in the deflection of underlying QNPA via FIB technique. We promise that this technique can be powerful tools for evaluation of the CTF distribution on the nanopatterned substrates.

## Competing interests

The authors declare that they have no competing interests.

## Authors’ contributions

DJK and GSK carried out the synthesis of nanostructures including silicon nanowires and quartz nanopillars and fluorescence measurements. DJK also prepared the samples for the SEM measurements and part of the drafted manuscript. GSK worked on the fluorescence measurements and helped to incubate the cells for the most time. JHH and WYL worked and analyzed cell traction force using FEM-based COMSOL software. CHH provided part of the financial support for this work. SKL organized all experiments and prepared most of the data and final manuscript. All authors read and approved the final manuscript.
